# Optimized Classification of Suspended Particles in Seawater by Dense Sampling of Polarized Light Pulses

**DOI:** 10.3390/s21217344

**Published:** 2021-11-04

**Authors:** Zhiming Guo, Hanbo Deng, Jiajin Li, Ran Liao, Hui Ma

**Affiliations:** 1 Institute for Ocean Engineering, Shenzhen International Graduate School, Tsinghua University, Shenzhen 518055, China; gzm20@mails.tsinghua.edu.cn (Z.G.); dhb19@mails.tsinghua.edu.cn (H.D.); lijiajin21@mails.tsinghua.edu.cn (J.L.); 2 Department of Biomedical Engineering, Tsinghua University, Beijing 100084, China; 3 Guangdong Research Center of Polarization Imaging and Measurement Engineering Technology, Shenzhen International Graduate School, Tsinghua University, Shenzhen 518055, China; mahui@tsinghua.edu.cn; 4 Shenzhen Key Laboratory of Marine IntelliSensing and Computation, Shenzhen International Graduate School, Tsinghua University, Shenzhen 518055, China; 5 Department of Physics, Tsinghua University, Beijing 100084, China

**Keywords:** classification, suspended particles, polarized light pulses, dense sampling

## Abstract

Suspended particles affect the state and vitality of the marine ecosystem. In situ probing and accurately classifying the suspended particles in seawater have an important impact on ecological research and environmental monitoring. Individual measurement of the optical polarization parameters scattered by the suspended particles has been proven to be a powerful tool to classify the particulate compositions in seawater. In previous works, the temporal polarized light pulses are sampled and averaged to evaluate the polarization parameters. In this paper, a method based on dense sampling of polarized light pulses is proposed and the experimental setup is built. The experimental results show that the dense sampling method optimizes the classification and increases the average accuracy by at least 16% than the average method. We demonstrate the feasibility of dense sampling method by classifying the multiple types of particles in mixed suspensions and show its excellent generalization ability by multi-classification of the particles. Additional analysis indicates that the dense sampling method basically takes advantage of the high-quality polarization parameters to optimize the classification performance. The above results suggest that the proposed dense sampling method has the potential to probe the suspended particles in seawater in red-tide early warning, as well as sediment and microplastics monitoring.

## 1. Introduction

The ocean is the most important resource endowed by nature, which contains the abundant resources necessary for the survival and development of human society [1]. While the ocean continues to create huge benefits for modern society, its own ecology has also encountered great challenges to human life and production [2,3,4]. As an important and essential component in seawater, the suspended particles significantly influence the optical properties of seawater, as well as the marine ecological environment [5,6]. For example, certain harmful types of microalgae rapidly cause algae blooms in a short period, which is a threat to marine organisms [7,8,9]. Microplastic has become a prevalent, widespread element of marine litter, threatening marine organisms and human health [10,11,12]. The accumulation and transportation of sediments such as silts, have a great impact on the stability of the estuarine and seacoast [13,14]. Therefore, the development of effective detection and accurate classification and identification of these different suspended particles is of far-reaching significance [15,16]. At the same time, the detected particle information is also helpful to interpret the data of marine science macroscopic researches, and further, promote the development of remote sensing in marine monitoring [17,18].

Optical methods are currently one of the most popular methods to detect the suspended particles, due to the advantages of high resolution, non-contact, and rich information [19,20]. In recent years, many in situ optical instruments have been applied to the acquisition of suspended particles in seawater such as YSI EXO [21,22], AC-S [23], BB9 [24], and LISST-200X [25]. However, these methods use bulk measurement to obtain comprehensive information of suspended particles in seawater, and cannot obtain information on the morphology and internal structure of individual seaweed particles. The Flow cytometers, such as FlowCytobot, individually measure the scattered intensity and fluorescence or sometimes the images of the particles to classify the particles in seawater. However, its dependency on the pretreatment is based on the hydrodynamic focusing system, limiting its application in the seawater [26,27].

Polarization is the fundamental property of light. Compared with traditional optical methods, polarized light can carry richer information [28]. The polarization information differences can be utilized for the analysis and identification of biological tissue lesions [29,30], and for the identification and classification of atmospheric and marine particles [31,32]. However, the polarization parameters are affected by the comprehensive effects of particles, including size, refractive index, shape, morphological structure, and microstructure. In 2018, Wang et al. obtained the polarized pulses of temporal signals through an experimental setup, and the signal-to-noise ratio of the system was bigger than 5. Then, they used a low-pass filter to suppress the high-frequency noise and used a threshold limit to acquire the polarized pulse signal. Finally, all the samplings in each polarized light pulse are calculated as an average value (PLP-Ave). This method is applied to differentiate the suspended particles of different physical and microstructural properties, which is important for monitoring microalgae, microplastics, and silt concentrations [33]. In 2019, Liao et al. applied the PLP-Ave method and developed a new in situ prototype, whose ability to the classification of the suspended particles in seawater has been demonstrated by field deployments [34]. In 2020, Li et al. used the PLP-Ave method to probe the collapse and regeneration of the cyanobacterial gas vesicles exposed to different static pressures [20]. In addition, Wang et al. chose samples with distinctive microstructural features, and then conducted simulations and calculations to examine how these features affect the polarization of the scattered photons using the PLP-Ave method [35].

This paper will introduce a method for optimizing the classification of suspended particles in seawater by dense sampling of polarized light pulses. The laboratory experiment which is free of the pretreatment of the samples, preliminarily shows that this method has local optimal characteristics and significant classification performance. Through the dense sampling of polarized light pulses, the characteristic information of the suspended particles can be obtained comprehensively, and a large number of local effective characteristics of the suspended particles can be improved to achieve high accuracy. In practical applications, the PLP-All model is more accurate and flexible in the mixed experiment prediction. These results show that using the dense sampling of polarized light pulses is beneficial to the more accurate classification of suspended particles. In addition, it brings a good application method for identifying and classifying suspended particles in seawater.

## 2. Methods and Materials

### 2.1. Principle of the Experimental Setup

A polarized light scattering method for differentiating suspended particles of different physical and microstructural properties can detect and classify the suspended particles [33,35]. On this basis, we built an experimental setup using a similar principle, which is free from the pretreatment of the samples. It achieves the classification of suspended particles in seawater by individual particle measurement and machine learning algorithms.

The experimental setup includes four parts: Optical path, photoelectric convertor, analog-to-digital conversion, and the sampling system. The sampling system consists of a sample pool and a flow circulation system. The sample pool has one inlet and one outlet. In addition, the sample is pumped by a pumper to enter the sample pool through the inlet and leave the sample pool through the outlet by another pumper. The particles in the sample are suspended and pass through the scattering volume of the setup, whose polarization parameters can be efficiently and accurately measured.

**Figure 1:**
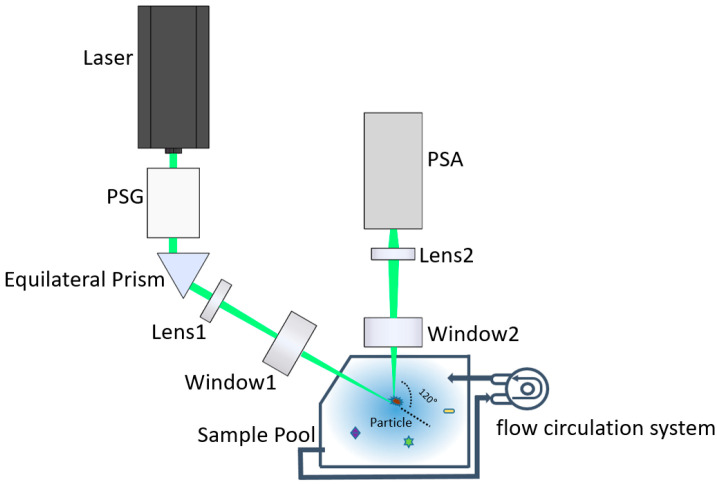
Schematic diagram of the optical system of the experimental setup.

Figure 1 shows the optical path of the setup. A 520 nm laser is the light source with 2-mm beam size and 0.7 W maximal power. Since most of the microalgae have a low absorption coefficient and high scattering coefficient at 520 nm [36], using the 520 nm laser the light is more conducive to reflecting the structure of microalgae and highlighting the differences between them. A polarization state generator (PSG) converts the light into the desired polarization states. The beam is completely reflected by the equilateral prism to obtain the obliquely illuminating light. Then, the illuminating light passes through Lens 1 and the transparent ceramic window 1, which is focused into a tiny light spot. Once the suspended particle passes through the light spot, it will be illuminated, and the 120° scattered light will pass through the transparent ceramic window 2 and be received by Lens 2. Note that windows 1 and 2 do not change the polarization states of the light passing through them. In addition, the 120° scattering angle has been proven to be sensitive to the microstructure in our previous work, which helps in identifying and distinguishing different suspended particles in seawater [35]. Then, the 120° scattering angle is used to maintain consistency with the previous work [33].

Lens 2 consists of a series of lenses and a pinhole. The size of the pinhole is 100-micron and its position is the imaging point of the light spot by the lenses before the pinhole. The pinhole is followed by a short focal length lens to convert the scattered light to the parallel light beam before entering the polarization state analyzer (PSA). Therefore, the scattering volume as the intersection volume of the illuminating optical path and the receiving optical path, is determined by the pinhole and the light spot. Moreover, in this work, the scattering volume is less than 0.01 microliter. If the volume concentration of the suspended particles is less than 10^5^ per milliliter, there is only one particle in the scattering volume at most, based on which the measurement of the individual particle can be realized. Therefore, the requirement for the volume concentration does not depend on the particle size, but depends on the scattering volume, which is determined by the pinhole and the light spot of the experimental device. In the measurement, if a single particle passes through the scattering volume, its scattered light contributes to the signal. When there are no particles in the scattering volume, the electronic noise, environmental light, and the scattering of water contribute to the background, which is smaller than the particle. Therefore, the signals are a series of temporal pulses.

PSG and PSA are the important components of the experimental setup, which realize the key functions of polarized light illumination and detection in the system. PSG is composed of the fixed linear polarizer, achromatic half-wave plate, and achromatic quarter-wave plate. In addition, the independent rotating motors carried by one-half wave plate and one-quarter wave plate change the direction of the fast axis of the wave plate. In our self-written application interface, the operator can set the rotation angle of the motor to obtain the desired polarization of the incident light, thereby obtaining more abundant polarization information of the particulate matter.

PSA is composed of three non-polarizing cube beam splitters, which divide the incident parallel beam into four parts. The two parts are analyzed with 0 and 45° linear polarizers and other analyzers. The other two parts are analyzed by left-hand and right-hand circular analyzers. The left-hand circular analyzer consists of a 135° fast-axis oriented quarter-wave plate and a 90° linear polarizer. In addition, the right-hand circular analyzer consists of a 45° fast-axis oriented quarter-wave plate and a 90° linear polarizer. 

Then, the 120° backward scattered light by the suspended particles in the scattering volume is divided into four channels by the PSA and converted into the four voltages by the four independent photoelectric converters. In addition, it is simultaneously digitized into a four-channel signal by a data acquisition card (DAQ). Thereafter, the four-channel signals are transferred into the polarization state of the scattered light by the instrument matrix gained from a polarization calibration procedure [34].

Using the experimental setup, we can measure the polarization state of the light scattered by the suspended particles and obtain a series of temporal pulses. Due to the individual measurement of the setup, each pulse originates from an individual particle. By processing these pulses, we can get the polarization parameters of suspended particles.

### 2.2. Samples

The suspended particles used in this experiment consist of five types of microalgae, two types of microplastics, and one type of sediment. The five types of microalgae (*Dunaliella salina* (DS), *Cryptomonassp.* (CP), *Chaetoceros debilis* (CD), *Phaeocystis globosa* (PG), and *Thalassiosira weissflogii* (TW)) were bought from Shanghai Guangyu Biological Technology Co., Ltd., which carried out a large-scale cultivation of liquid microalgae species based on the production environment including the temperature, nutrient salt formula, and light intensity. In particular, DS has a unique economic value in medicine and health care [37], CD is often used in research studies for oceanography and aquaculture [38], PG is toxic and will cause red tide [39], CP and TW are important components for the phytoplankton ecosystem and productivity [40,41].

In addition, other suspended particles (monodispersed polystyrene microspheres, 2 μm (PS-02) and 10 μm (PS-10), silicon dioxide pellets, 10 μm (SD-10)) were bought from Big Goose (Tianjin) Technology Co., Ltd. They are all white suspensions obtained by dispersing white solid powder in water. The SD-10 mother suspension was prepared by mixing 250 mg of silicon dioxide pellets in 10 mL of 50% ethanol water solution. Moreover, the PS-02 and PS-10 mother suspensions were prepared by mixing 250 mg of monodispersed polystyrene microspheres in 10 mL of deionized water solution.

Filtered seawater is used in this work. We obtained the surface seawater from Yantian Port in Shenzhen, and filtered it in the lab with a 0.2-micron filter membrane. The filtered seawater is prepared in advance to dilute the mother suspensions of the particles.

### 2.3. Polarized Light Pulse Processing Algorithm

**Figure 2:**
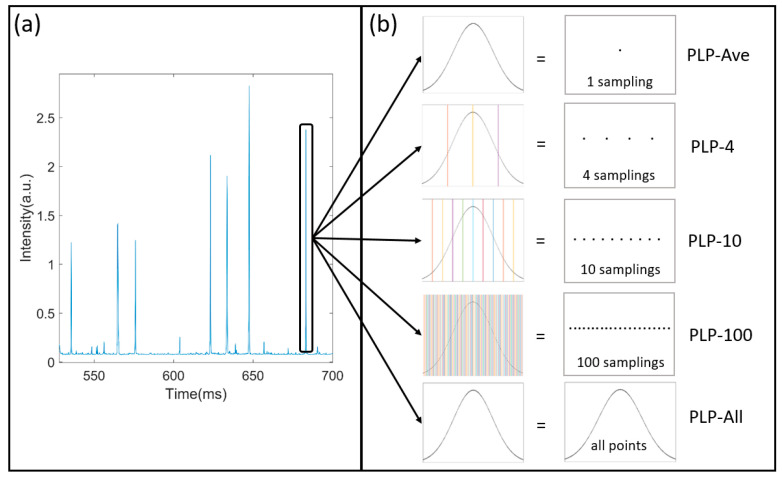
The dense sampling of polarized light pulses: (**a**) The temporal signals; (**b**) polarized light pulse processing algorithm.

Figure 2a shows the temporal signals of a suspension measured by the setup, which is a series of polarized light pulses. The envelope of pulses is obtained by the treatment of low-pass filtering. The polarized light pulses include the information of the particle and the noises. In addition, the filtering can reduce the noisy fluctuation that originated from the electronic noise or environmental noise. However, there is still residual noise in the pulses.

When a single suspended particle passes through the scattering volume, it stays for a while during which it is continuously illuminated and its scattered polarization states are measured. The width of the polarized light pulse is the settling time that the particle stays in the scattering volume. In this case, the particle would be sampled many times by the setup. For example, in Figure 2b, a pulse’s settling time is about 2 ms and if the sampling rate of the DAQ is 200 K sampling per second, then this pulse consists of 400 samplings and the particle is measured 400 times at one measurement. We have 400 scattered polarization states of the particles. Considering that the particle is moving in the scattering volume, we obtain abundant data of this particle and the method of extracting the information from these data is a serious issue. Due to the noises from the electronic system and the environmental light, in previous researches, we first averaged all of the samplings in each polarized light pulse (PLP-Ave) to reduce the influence of the noises on the signals before classifying the suspended particles.

In this work, we introduce the polarized light pulse processing algorithm and investigate the benefit or loss of the averaging of the polarized light pulses to achieve more efficient and accurate classification than before. We divide the polarized light pulses with specific methods into four samplings (PLP-4), 10 samplings (PLP-10), 100 samplings (PLP-100), and all points (PLP-All), as shown in Figure 2b. For example, in the PLP-4 method, we divide the polarized light pulses into four parts and average each part to obtain four values for one pulse, which is considered as four samplings. Similarly, we get 10 samplings for one pulse in the PLP-10 method, and 100 samplings in the PLP-100 method. For the PLP-All method, all the samplings are considered. Generally, the averaging will suppress the noise and enhance the signal-noise-ratio, and finally help in extracting the information from the polarized light pulse. However, the averaging will omit the detailed information. Definitely, the PLP-All method suffers the most from the noise and the values are most inaccurate. Therefore, this investigation will try to estimate the benefit and loss of the averaging of the pulses using the polarized light pulse processing method.

### 2.4. Analytical Methods

Stokes vector, *S*, as shown in Equation (1) is always used to describe the polarization state of light [42].

(1) $$S=\left[\begin{array}{c} I\\ Q\\ U\\ V \end{array}\right]$$

where *I* is the total light intensity, and *Q*, *U*, *V* are the residual 0°, 45°, and right-circularly polarization, respectively.

As shown in Equation (2), *q*, *u*, and *v* are polarization parameters normalized by light intensity *I*, which can be dimensionless and range from −1 to 1.

(2) $$q=\frac{Q}{I},\;u=\frac{U}{I},\;v=\frac{V}{I}.$$

The degree of polarization (DOP) as shown in Equation (3) commonly represents the proportion of polarized light in the total light intensity, ranging from 0 to 1, which is also used to characterize the depolarization ability of particles when they are illuminated by a polarized light [43].

(3) $$\mathrm{DOP}=\frac{\sqrt{Q^{2}+U^{2}+V^{2}}}{I}$$

For classification problems, the results of machine predictions and actual values will deviate. The confusion matrix is a standard measure that represents accuracy evaluation, including true positive (TP), false positive (FP), true negative (TN), and false negative (FP) [44]. Each row of this matrix represents an instance in the actual class, and each column represents an instance in the predicted class. It can clearly express the correct classification and misclassification of each category on the visual system. Therefore, the evaluation model’s advantages and disadvantages standards introduce accuracy, which is computed from the confusion matrix using Equation (4).

(4) $$A\mathrm{ccuracy}=\frac{TP+TN}{TP+FP+FN+TN}$$

where TP denotes that the positive class is judged as a positive class, FP denotes that the negative class is judged as a positive class, TN denotes that the positive class is judged as a negative class, and FN denotes that the negative class is judged as a negative class.

As shown in Equation (5), the mean-square error (*MSE*) is a measure that reflects the degree of difference between the estimator and the true value. $\hat{x}$ is the estimator of the sample, and *x* is the true value of the sample. When the sample is constant, their distance function is an index used to evaluate the quality of an estimator.

(5) $$MSE(\hat{x})=E{\left(\hat{x}-x\right)}^{2}$$

### 2.5. Algorithm Theory

**Figure 3:**
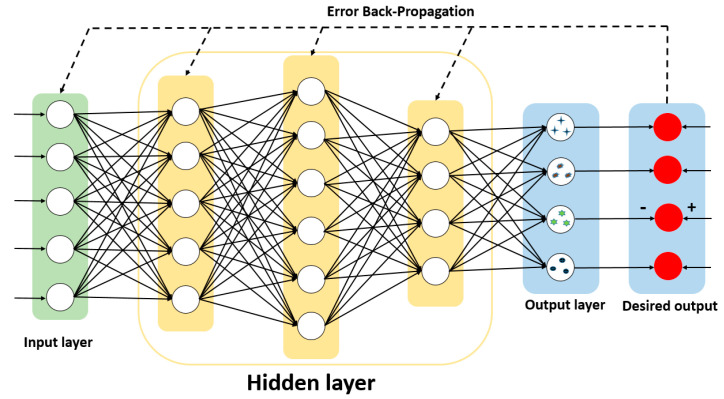
The schematic diagram of backpropagation algorithm structure.

As one of the most traditional neural networks, the main characteristic of backpropagation neural network (BPNN) is that the signal propagates forward and the error propagates backward. Backpropagation is the standard method for training artificial neural networks. This method helps in calculating the gradient of the loss function with respect to all the weights in the network. By fine-tuning the weight of the neural network based on the error rate obtained in the previous period, it can reduce the error rate and improve the generalization and reliability of the model. Therefore, the training and test sets of the BPNN algorithm model that we built use the polarization parameter data, which is measured by the suspended particles in the experimental device. As shown in Figure 3, the input layer of the network training uses X=[I,q,u,v, DOP], and there are five input nodes. Through multiple experiments, the hidden layer is set to three layers, which are five nodes, six nodes, and four nodes. Finally, there are four nodes in the output layer, corresponding to four types of suspended particles. In addition, we built the algorithm model using the sigmoid nonlinear transfer function, which minimizes the actual output and expected output error function of the system by revising weights and thresholds repeatedly.

## 3. Results

### 3.1. Classification of the Four Types of Microalgae

In this paper, we built the BPNN model which is used to classify the four types of microalgae, DS, CP, CD, and PG for each method of the polarized light pulse process algorithm. The dataset prepared by the PLP-Ave method is used as input data for training and testing and finally, for building the PLP-Ave model. Similarly, we built the PLP-4 model, PLP-10 model, PLP-100 model, and PLP-All model. For each model, the total number of dataset is 11,000. We used the random function of MATLAB to sort the dataset of each model. Then, 70% of the disordered dataset is used for training and 30% is used for testing.

**Figure 4:**
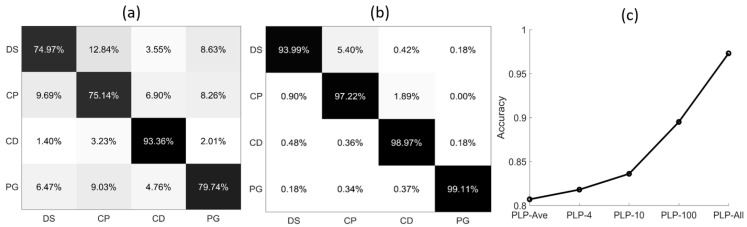
The confusion matrix of different polarized light pulses methods: (**a**) PLP-Ave model; (**b**) PLP-All model; (**c**) average accuracy of five models.

After training for 100 epochs, the model tends to converge and the results are shown in Figure 4. Figure 4a shows the confusion matrix of PLP-Ave model. As can be seen, the classification accuracy for the DS, CP, and PG are less than 80%, and some errors are around 10%. The average accuracy of PLP-Ave model is only 80.77%. In Figure 4b, the classification accuracy of PLP-All model for all the types of microalgae is larger than 90%, and for PG it is larger than 99%. The average accuracy of PLP-All model is 97.32%. Next, we collect the average accuracy of the different methods in Figure 4c. As can be seen, the PLP-All model has achieved the best classification accuracy, and the PLP-Ave model’s accuracy is minimal. In addition, the classification accuracy for the suspended particles increases with the sampling numbers in the different methods. Note that the PLP-All model suffers the most from the noises in the data. However, since it contains most of the information about the particles, it achieves the best classification accuracy.

The classification accuracy of the four types of microalgae in general is shown in Table 1. In this case, the classification accuracy of the model trained by the data of PLP-All model is larger by 16.55% than the PLP-Ave model, which indicates that this model has stronger feature extraction capabilities. These high-precision results are consistent in both the training and prediction sets, which also prove that the model we trained has a strong generalization ability.

### 3.2. The Mixed Experiment Prediction

To check the flexibility and feasibility of the above models, we used them to classify the four types of microalgae cells in the mixed suspensions. First, we measured the four types of microalgae suspensions separately. In order to collect enough particles, we added 2 milliliter of DS to the filtered seawater and measured for 3 min, then recorded the resulting pulse number which is 720 pulses. Similarly, the same steps were performed for CP, CD, and PG, respectively. In addition, we obtained the polarized light pulse numbers, 2160 pulses for CP, 576 pulses for CD, and 648 pulses for PG. Then, we mixed the four microalgae suspensions together, Groups 1, 2, and 3. In each group, the respective volumes were carefully chosen to ensure that the obtained pulse number by the setup for each type of microalgae was consistent with the preset pulse number. Then, the mixed suspensions were separately measured by the experimental setup and each measurement lasted 3 min. The obtained data were fed into the PLP-Ave and PLP-All models respectively to predict the number of microalgae cells in each group. For the pulses of temporal signals information, we need to accumulate enough pulses to obtain most of the particle information. In addition, the machine learning of BPNN algorithm needs enough characteristics of particles in order to correctly identify the category of particles.

Table 2 collects the preset pulse number by the calculated volume and the predicted pulse number by the models for the three mixed suspensions, which can be regarded respectively as the true value and the predicted value by the models. There are obvious differences between the preset pulse numbers and the predicted pulse numbers gained by the PLP-Ave model. The largest error of PLP-Ave model occurs at Group 1, the predicted pulse number for DS is 100, while the preset pulse number is 40, and the relative error is about 60%. However, the largest error of PLP-All model occurs in the same group, the predicted pulse number for DS is 100, while the preset pulse number is 75, and the relative error is about 25%. Generally, the errors between the preset and predicted pulse numbers of PLP-All model are much smaller than the PLP-Ave model. Note that the shape and microstructure of some specific microalgae are not obvious, and an excessive increase in number cannot be well identified by the PLP-All model. Therefore, some specific type of the microalgae worsens the errors of the PLP-All model. However, most other types of the microalgae are good and acceptable, which are quite different from the PLP-Ave model. The results indicate that the PLP-All model is flexible and feasible for use in the classification of the microalgae in mixed suspensions.

### 3.3. Comparative Analysis

**Figure 5:**
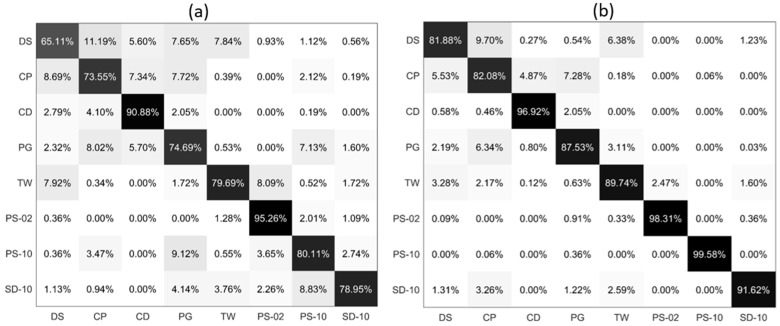
The confusion matrix of eight suspended particles classification: (**a**) PLP-Ave model; (**b**) PLP-All model.

Based on the classification performance of the PLP-All model, we tested a diverse particle group that consists of five types of microalgae, two types of microplastics, and one type of sediment, and the results are shown in Figure 5. Since TW and PG can both cause a red tide, we added TW into the group to confirm that the PLP-All method has a strong generalization ability and strong feature extraction ability. Through the confusion matrix of PLP-Ave model in Figure 5a, it can be seen that the highest classification accuracy for the eight suspended particles is less than 80%, and the lowest classification accuracy is 65.11%. The average accuracy of PLP-Ave model is only 80.20%. In Figure 5b, the classification accuracy of PLP-All model for all types of the suspended particles is larger than 81%, and for PS-10 it is larger than 99%. The average accuracy of PLP-All model is 90.90%. In addition, we noticed that all the suspended particles classification accuracy increase and the maximal increase in the accuracy is about 19.47%. Moreover, for PS-02, which has a rather high accuracy, the PLP-All model still improves the classification accuracy. Meanwhile, for the total effects, half of the particle’s classification accuracy increases by more than 10%.

The classification accuracy of the eight suspended particles in the training and test sets using the PLP-Ave and PLP-All models are shown in Table 3. The model’s classification accuracy which is trained by the data of the PLP-All model is larger by 10.70% than the PLP-Ave model, which indicates that this model has stronger feature extraction capabilities.

In general, for the diverse particles classification results in Figure 5, as compared with the PLP-Ave model, the PLP-All model effectively improves the classification accuracy. These results show that the PLP-All model has a strong generalization ability and impressive classification performance.

## 4. Discussion

### 4.1. Training Details of Different Models

**Figure 6:**
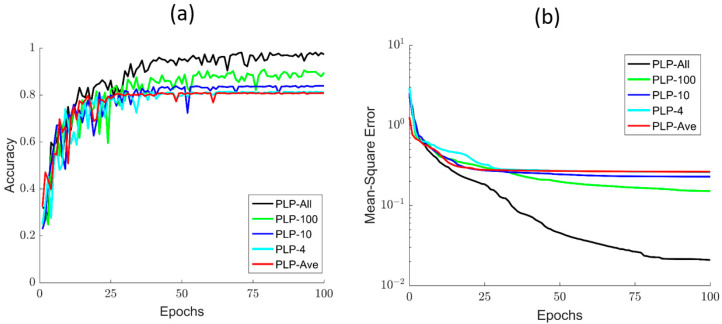
(**a**) The accuracy curve of different processing methods; (**b**) the MSE of different processing methods.

To further show the classification effects of the polarized light pulse processing algorithm, we provide the training details of all the models for different parts. As the number of epoch increases, the accuracy and MSE of these different models are evidently different, as shown in Figure 6a,b. Of note, when the number of epoch is less than 30, all the models increase sharply, but fluctuate strongly and show little differences in accuracy when compared with each other. However, as the number of epoch increases by more than 40, the PLP-Ave model converges to the stable value at first, but then its accuracy becomes worse. On the contrary, the accuracy performance of the PLP-All model is better than the other four models, but converges most slowly. Meanwhile, we can easily see that the models’ accuracy increases with the sampling number of the polarized light pulse. Moreover, these models still have a large difference in MSE. The MSE of PLP-All model reduces the fastest and reaches a lower value of about 0.05. In addition, after the MSE’s stable value reduces with the sampling number, these training details indicate that the PLP-All method has better classification ability, as shown in Figure 6. All of these results emphasize that the PLP-All method can effectively distinguish the CD algae with large differences in the microstructure. In addition, the PLP-All method can still distinguish the microalgal samples (DS, CP, PG) with little differences in appearance and structure. This is related to the effect of polarization information on size, shape, microstructure, and morphology, in order to extract high-quality data from the polarization pulses.

### 4.2. Accuracy of Four Microalgal Samples

**Figure 7:**
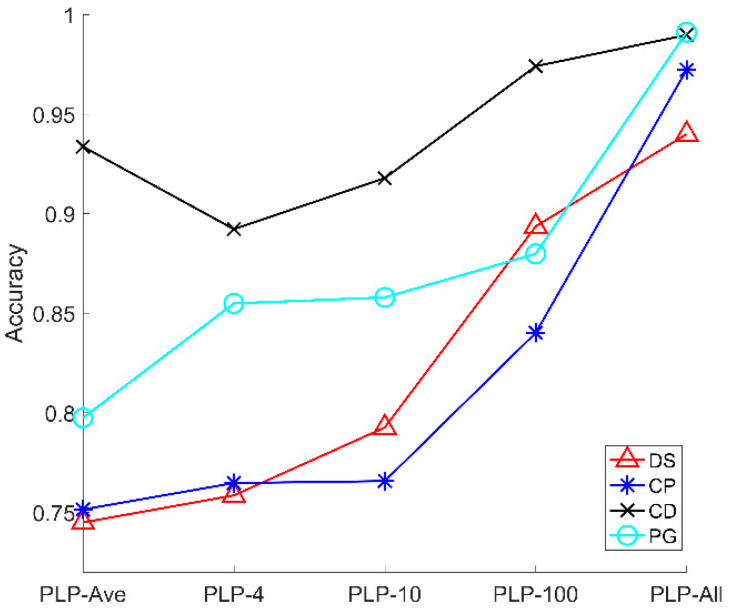
The average accuracy of five polarized light pulses processing methods for signal particles.

Figure 4c shows the average accuracy of five models. Here, as shown in Figure 6, we provide the accuracy of five models for four microalgal samples. From Figure 7, the accuracy of the three types of microalgae (DS, CP, PG) increases with the sampling number of the polarized light pulse, which is consistent with the average accuracy in Figure 4c. However, the CD is a little different, and its accuracy increases when the sampling number grows from 4 to 100, and finally reaches the maximum in the PLP-All model. In addition, the accuracy of PLP-Ave is larger than PLP-4 and PLP-10, which is quite different from the other models. Therefore, we notice that the accuracy of CD samples is larger than the other microalgal samples. In addition, the accuracy changes in a different manner than the models for the four samples.

Essentially, the accuracy of models for the microalgal samples is subjected to the sensitivity of the polarization parameters to the physical properties of the microalgae cells. The CD cells have flagella and their shape are long oval, which is quite different from the other microalgae. This specific microstructure contributes to the higher accuracy of CD at all the models than the other microalgae.

In addition, we should recall that the averaging in the polarized light pulse will reduce the noise and then, lead to the accurate measurement of the polarization parameters. However, the samplings of the polarized light pulse will increase the information amount, which are both positive for the classification results. When we divide the polarized light pulse in 4, 10, and 100 times or take all the sampling points of the polarized light pulse into account, we suffer more from the noise but increase the information amount of the microalgae cells. As a result, the classification accuracy would be the tradeoff between these two factors. For CD, averaging plays a more important role at first, thus the accuracy of PLP-Ave is larger than PLP-4 and PLP-10, but then the information amount increases and dominates the classification. Therefore, the accuracy of PLP-100 and PLP-All is larger than PLP-Ave. Moreover, the inherent difference between the microalgal types and the tradeoff between the averaging and information amount determine the classification accuracy of the models.

### 4.3. Origin of the Performance of PLP-All Method

**Figure 8:**
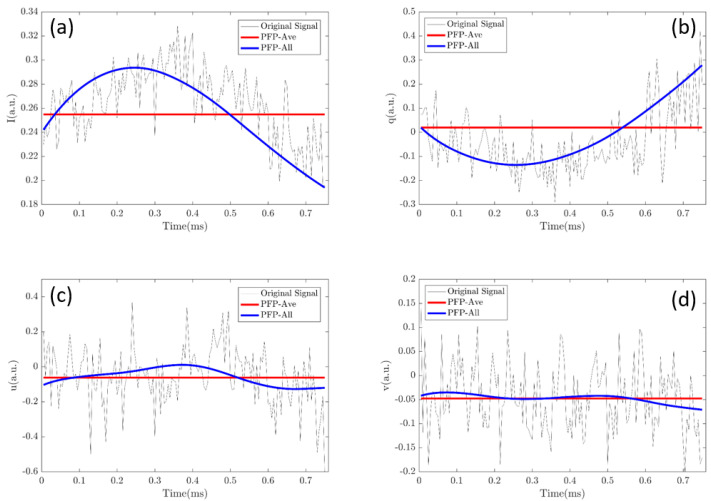
Temporal values of a polarized light pulse processed by the PLP-All method and the dash line is the value given by the PLP-Ave method: (**a**) Intensity values (I); (**b**) polarization parameter (q); (**c**) polarization parameter (u); (**d**) polarization parameter (v).

Of note, the PLP-All method suffers the most from the noise of the data, but the classification accuracy is the best. Evidently, the increase of the data amount is one origin for the best performance. In addition, in Figure 4c, it can be seen that increasing the data amount will promote the accuracy. However, we would like to emphasize another origin, which is possibly more essential than the amount of data. Figure 8 shows part of the data, [*I*, *q*, *u*, *v*], of a polarized light pulse processed by the PLP-Ave and PLP-All methods, respectively and their original signal. The red lines are the average values given by the PLP-Ave method and the blue lines are the temporal values given by the PLP-All method. The temporal values change in the polarized light pulse. Here, we can imagine that if we use the average values given by the PLP-Ave method to replace those of the PLP-All model, the same data amount is ensured, but the classification effect would be no better than the PLP-Ave model. Therefore, the key origin of the best performance of PLP-All method is implied by the time-changing values of polarization parameters of the individual particle, as shown in Figure 8.

Moreover, Figure 8 shows the original signals in the pulse, which are noisy and not filtered by the low-pass filter. If we use these original signals to feed the classifier, the data amount is the same, but the final classification effect is the worst. Although the entire information of the particle is in the original signal, the noises destroy the classification ability. The envelope of the pulse gained by the low-pass filtering in Figure 2 and Figure 8 possibly loses some particle information, but it reduces the noise, which indicates that the PLP-All method takes advantage of the denoised and high-quality data.

For each suspended particle crossing the scattering volume, the transient location and orientation change with the time, according to the setup’s optical system, which leads to the changes of their scattered polarization states and parameters. Note that the polarization parameters are sensitive to the structures and orientation of the particles. Therefore, they can well characterize this essential information, and finally provide high-quality data. The dense sampling of each pulse records these transient states of the individual particle, which enhances the information collection. In summary, the PLP-All method takes advantage of the polarization parameters and dense sampling, and then achieves the best performance.

In addition, compared with the existing classification methods of suspended particles, the method proposed in this paper has the advantage of not requiring a sample pretreatment and the ability of detecting the rich polarization information of the individual particles. However, the time efficiency needs to be enhanced in the next step. Moreover, beyond the current Stokes vector measurement, the Muller matrix measurement of the individually suspended particles may be added to the future worklist.

### 4.4. Comparative Different Machine Learning Algorithms

To further confirm that the different polarized light pulse methods for particle classification depend little on the machine learning algorithm, we additionally built the support vector machine (SVM) algorithm. SVM was used to classify the four types of microalgae, DS, CP, CD, and PG for different polarized light pulse process methods and the results are shown in Table 4. A comparison of Table 1 and Table 4 showed that the classification accuracy of the PLP-Ave model using the BPNN algorithm was larger by about 6% than the SVM algorithm. Similarly, the accuracy of PLP-All model was larger by about 3% than the SVM algorithm. This indicates that the BPNN algorithm was more powerful in extracting the polarization parameter features for achieving higher accuracy than the SVM algorithm. In addition, we would like to emphasize that the different machine learning algorithms led to the similarly excellent performance of PLP-All method for particle classification.

## 5. Conclusions

In this paper, we proposed an optimization method for the classification of suspended particles in seawater by dense sampling of polarized light pulses. We built an experimental setup to measure the suspended particles and collect the polarized light pulses. Then, we investigated the classification results of the four types of microalgae using different dense sampling methods. For each method, we sampled the pulse with a certain number and then built the specific model to classify the four types of microalgae. The results showed that the classification accuracy increased with the sampling numbers. In addition, the PLP-All model achieved the best classification performance. Moreover, we classified the four types of microalgae cells in the mixed suspensions and the results indicated that the PLP-All model was feasible. Furthermore, we conducted eight types of suspended particles including microalgae, microplastics, and sediment, and the classification results showed that the PLP-All model had a good generalization ability. In the discussion part, the classification accuracy increased with the sampling number, but the MSE decreased. In addition, for each type of microalgae, the PLP-All model was still the best and the dense sampling improved the classification performance. Finally, the best performance of the PLP-All model can be attributed to taking advantage of the high-quality polarization parameters and dense sampling. In summary, the method based on dense sampling of polarized light pulses had an excellent ability of classifying the suspended particles. It can be expected that the underwater polarization scattering instrument equipped with dense sampling can effectively and accurately help in obtaining the information of particle compositions in seawater. Furthermore, the dense sampling idea can be used in the future development of Muller matrix polarimetry of the suspended particles.

## Figures and Tables

**Table 1:** Accuracy of PLP-Ave model and PLP-All model.

Dataset	Training Set	Test Set
PLP-Ave model	80.87%	80.77%
PLP-All model	97.80%	97.32%

**Table 2:** The predicted results of mixed suspensions.

	Preset Pulse Number	PLP-Ave Model Prediction	PLP-All Model Prediction
Group 1	100, 100, 100, 100	115, 104, 133, 40	115, 103, 99, 75
Group 2	200, 300, 100, 200	292, 258, 80, 194	214, 308, 108, 194
Group 3	200, 400, 100, 300	280, 320, 84, 286	234, 364, 100, 268

**Table 3:** Accuracy of PLP-Ave model and PLP-All model.

Dataset	Training Set	Test Set
PLP-Ave model	79.89%	80.20%
PLP-All model	90.80%	90.90%

**Table 4:** Accuracy of PLP-Ave and PLP-All models using the SVM algorithm.

Dataset	Training Set	Test Set
PLP-Ave model	74.39%	74.38%
PLP-All model	94.71%	94.57%

## Data Availability

We did not report any data.
